# Positive Evolutionary Selection On the RIG-I-Like Receptor Genes in Mammals

**DOI:** 10.1371/journal.pone.0081864

**Published:** 2013-11-27

**Authors:** Ana Lemos de Matos, Grant McFadden, Pedro J. Esteves

**Affiliations:** 1 CIBIO - Centro de Investigação em Biodiversidade e Recursos Genéticos/InBio Laboratório Associado, Universidade do Porto, Vairão, Portugal; 2 Departamento de Biologia, Faculdade de Ciências, Universidade do Porto, Porto, Portugal; 3 Department of Molecular Genetics and Microbiology, University of Florida, Gainesville, Florida, United States of America; 4 CESPU, Instituto de Investigação e Formação Avançada em Ciências e Tecnologias da Saúde, Gandra, Portugal; Boston College, United States of America

## Abstract

The mammalian RIG-I-like receptors, RIG-I, MDA5 and LGP2, are a family of DExD/H box RNA helicases responsible for the cytoplasmic detection of viral RNA. These receptors detect a variety of RNA viruses, or DNA viruses that express unusual RNA species, many of which are responsible for a great number of severe and lethal diseases. Host innate sentinel proteins involved in pathogen recognition must rapidly evolve in a dynamic arms race with pathogens, and thus are subjected to long-term positive selection pressures to avoid potential infections. Using six codon-based Maximum Likelihood methods, we were able to identify specific codons under positive selection in each of these three genes. The highest number of positively selected codons was detected in MDA5, but a great percentage of these codons were located outside of the currently defined protein domains for MDA5, which likely reflects the imposition of both functional and structural constraints. Additionally, our results support LGP2 as being the least prone to evolutionary change, since the lowest number of codons under selection was observed for this gene. On the other hand, the preponderance of positively selected codons for RIG-I were detected in known protein functional domains, suggesting that pressure has been imposed by the vast number of viruses that are recognized by this RNA helicase. Furthermore, the RIG-I repressor domain, the region responsible for recognizing and binding to its RNA substrates, exhibited the strongest evidence of selective pressures. Branch-site analyses were performed and several species branches on the three receptor gene trees showed evidence of episodic positive selection. In conclusion, by looking for evidence of positive evolutionary selection on mammalian RIG-I-like receptor genes, we propose that a multitude of viruses have crafted the receptors biological function in host defense, specifically for the RIG-I gene, contributing to the innate species-specific resistance/susceptibility to diverse viral pathogens.

## Introduction

The mammalian innate immune system operates as the first line of defense against microbial pathogen invasion [1-3]. This system recognizes infectious agents through a limited number of germline-encoded pattern-recognition receptors (PRRs) predominantly expressed on sentinel cells [2,4-6]. The host PRRs recognize and react with specific microbial components, known as pathogen-associated molecular patterns (PAMPs), which includes bacterial lipopolysaccharides, peptidoglycans, lipoteichoic acids and cell-wall lipoproteins, fungal β-glucan and viral nucleic acids [2,3,5,6]. The host PRRs exhibit distinct expression patterns and following sensing of their cognate ligands, activate specific signaling pathways that lead to the expression of a variety of inducible self-defense genes involved in the collective inflammatory and immune responses [2]. To date, four different classes of PRRs have been identified, including the cell membrane-associated C-type lectin receptors (CLRs), the Toll-like receptors (TLRs) at the cell surface and at the membrane of intracellular vesicles (endosomes and lysosomes), and the cytoplasmic detection systems for intracellular PAMPs, namely the RIG-I-like receptors (RLRs) and the NOD-like receptors (NLRs) [3,6-8].

The RLRs are a family of DExD/H box RNA helicases critically and exclusively involved in the recognition of “nonself” RNA from actively replicating viruses in the cytoplasm of infected cells [9]. This receptor family consists of three members, the retinoic acid-inducible gene-I (RIG-I/DDX58), the melanoma differentiation associated factor 5 (MDA5/IFIH1) and the laboratory of genetics and physiology 2 (LGP2/DHX58) [10-14]. RIG-I and MDA5 share high sequence similarity and several structural features, including an N-terminal region consisting of tandem caspase activation and recruitment domains (CARDs), a central DExD/H box RNA helicase domain and a C-terminal domain (CTD). The two N-terminally located CARDs function as a signaling and interaction domain with other CARD-containing proteins [13,15,16]. The helicase domain retains catalytic activity to bind and unwind double stranded RNA (dsRNA) in an ATP hydrolysis-dependent manner [10,17]. The CTD plays a predominant role in high-affinity binding with dsRNA, encoding a repressor domain (RD) in RIG-I, but not in MDA5, which harbors an RD-like domain that does not participate in autoregulation [18]. These two RLRs detect a variety of both DNA and RNA viruses, particularly at early phase of viral replication, and signal the production of type I interferons (IFNs) and induction of an anti-viral response [10,17]. The third element of the RLR family, the LGP2 protein, lacks any CARDs but contains the helicase domain and the CTD also harbors a RD. The role of LGP2 in anti-viral immunity is less clear, but it has been suggested in different studies to serve both as a negative and a positive regulator of RIG-I and MDA5 signaling [10,19-21].

Despite the similarities between RIG-I and MDA5, they were shown to play different roles in anti-viral immunity by recognizing and protecting from specific classes of viruses [22]. RIG-I detects preferentially and most effectively short RNA sequences marked with 5’-triphosphate group (5’-ppp) and blunt-end of short double-stranded RNAs (dsRNAs) or single-stranded RNA (ssRNA) hairpins [23-27]. As a key sensor of ssRNA viruses, RIG-I is implicated in the response to Arenaviridae [28], Bunyaviridae [28], Filoviridae [28], Flaviviridae [18,29], Orthomyxoviridae [22,30], Paramyxoviridae [22,28,30,31] and Rhabdoviridae [22,23]. On the other hand, MDA5 is activated by high-molecular-weight poly(I:C) fragments [22,32], and also by long-duplex RNAs from the genomes of dsRNA viruses [30] or dsRNA replication intermediates of positive-strand viruses, such as Caliciviridae [33], Coronaviridae [34] and Picornaviridae [22,32]. Regardless the virus recognition specificity by RIG-I and MDA5, some viruses are redundantly sensed by both RLRs, such as the West Nile virus and the Dengue virus [30,35]. In addition to the extensively described recognition of RNA viruses by RIG-I and MDA5, a role in anti-viral signaling in response to several dsDNA viruses has also been observed. As an RNA sensor, RIG-I does not detect DNA directly; however, not only do many DNA viruses create dsRNA products by virtue of convergent transcriptional units derived from opposite strands, but also the host RNA polymerase III can mediate the transcription of cytoplasmic DNA templates (such as transfected poly dA:dT) into dsRNA containing 5’-triphosphate, which will activate RIG-I and trigger the production of type I IFN [36,37]. Both Epstein-Barr virus and myxoma virus are detected by RIG-I, while vaccinia virus is sensed by MDA5 [38-40]. It is also likely that the precise RLRs utilized for the sensing of specific viruses also operate within cell-specific contexts as well.

Interaction between host and pathogen results in a dynamic arms race. Whenever pathogens develop strategies to overtake the host immune system, the host proteins involved in pathogen recognition have to respond by evolving to avoid or reduce potential infections. These dynamics result in host-pathogen adaptation and counter-adaptation, which in turns lead to the rapid co-evolution of both parties. Particularly for the host, this accelerated molecular evolution is often reflected in host defense genes that exhibit strong signatures of ongoing diversifying selection [41,42]. Because viruses are responsible for a great number of severe and lethal diseases, together with the important role that RLRs play in mammalian innate immune system, we expect that RIG-I, MDA5 and LGP2 genes may have been under intense selective pressures in all mammals. We have previously demonstrated that one other class of mammalian PRRs, the TLRs, exhibit striking evidence of positive genetic selection as a result of selective pressures exerted by pathogens [43]. Using six different codon-based Maximum Likelihood (ML) methods, we searched for evidence of long-term selective pressures in the three RLR genes present in the available sequenced mammalian genomes and, where possible, pinpoint positively selected residues that might be involved in the host-virus interactions that have shaped their rapid diversification. Specific lineages subject to episodic positive selection have also been identified in the three RLR genes by using two different branch-site models.

## Results

### Mammalian RIG-I-like receptor gene sequences

Publicly available mammalian RIG-I, MDA5 and LGP2 gene sequences were collected from Ensembl and NCBI databases (Table S1) for phylogenetic and selection analyses. The nucleotide coding sequences for each of the three RLR gene orthologous were aligned and are represented in Figure S1 (RIG-I alignment), Figure S2 (MDA5 alignment) and Figure S3 (LGP2 alignment). The translation into deduced protein sequences is also represented in Figure S4 (RIG-I alignment), Figure S5 (MDA5 alignment) and Figure S6 (LGP2 alignment).

The inherent limitations of using solely publicly available mammalian RLRs sequences should be highlighted, although several studies have used the same source of data for general conclusions about other genes in mammals [43-49]. The analyses performed ahead use only an individual representative of each included species and therefore, any drawn conclusions should be carefully considered. 

### Phylogenetic reconstruction of mammalian RIG-I-like receptors

ML trees were reconstructed for RIG-I, MDA5 and LGP2 genes after looking for evidence of recombination using the software GARD [50,51] implemented in the Datamonkey web server [52,53].

**Figure 1:**
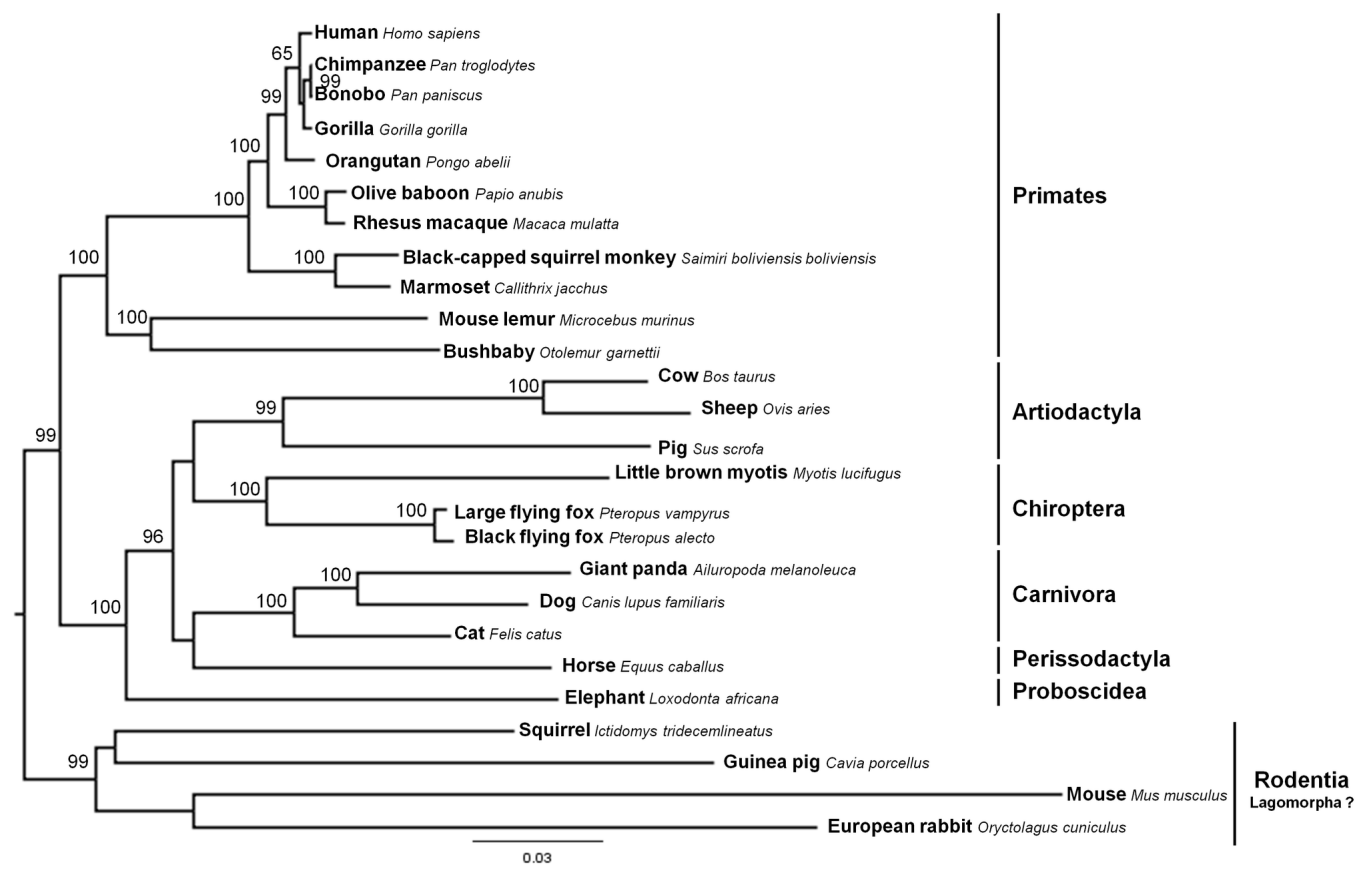
Maximum likelihood (ML) phylogenetic tree of RIG-I gene used for codon-based ML analysis. The GTR+G nucleotide substitution model was employed in mammalian RIG-I gene phylogenetic reconstruction. Bootstrap values >50 are indicated on the branches.

In mammalian RIG-I phylogenetic reconstruction, the monophyly of six of a total of eight taxonomic orders was observed (Figure 1). However, an interesting fact was registered for the two remaining orders, Rodentia and Lagomorpha, when the European rabbit (order Lagomorpha) grouped with the rodent cluster. When looking carefully at the European rabbit RIG-I deduced protein sequence (Figure S4), a great number of conserved regions between this species and the other mammalian species was observed, with the exception of a region between codons 840 and 879. This 40 amino acid domain region was unique to the European rabbit RIG-I. We originally speculated that this difference might have been the result of a gene conversion event with adjacent genes. However, when we examined the genes that are chromosomally adjacent to European rabbit RIG-I (NDUFB6, TOPORS and FRP), no clear evidence of gene conversion was detected by the software GARD [50,51].

**Figure 2:**
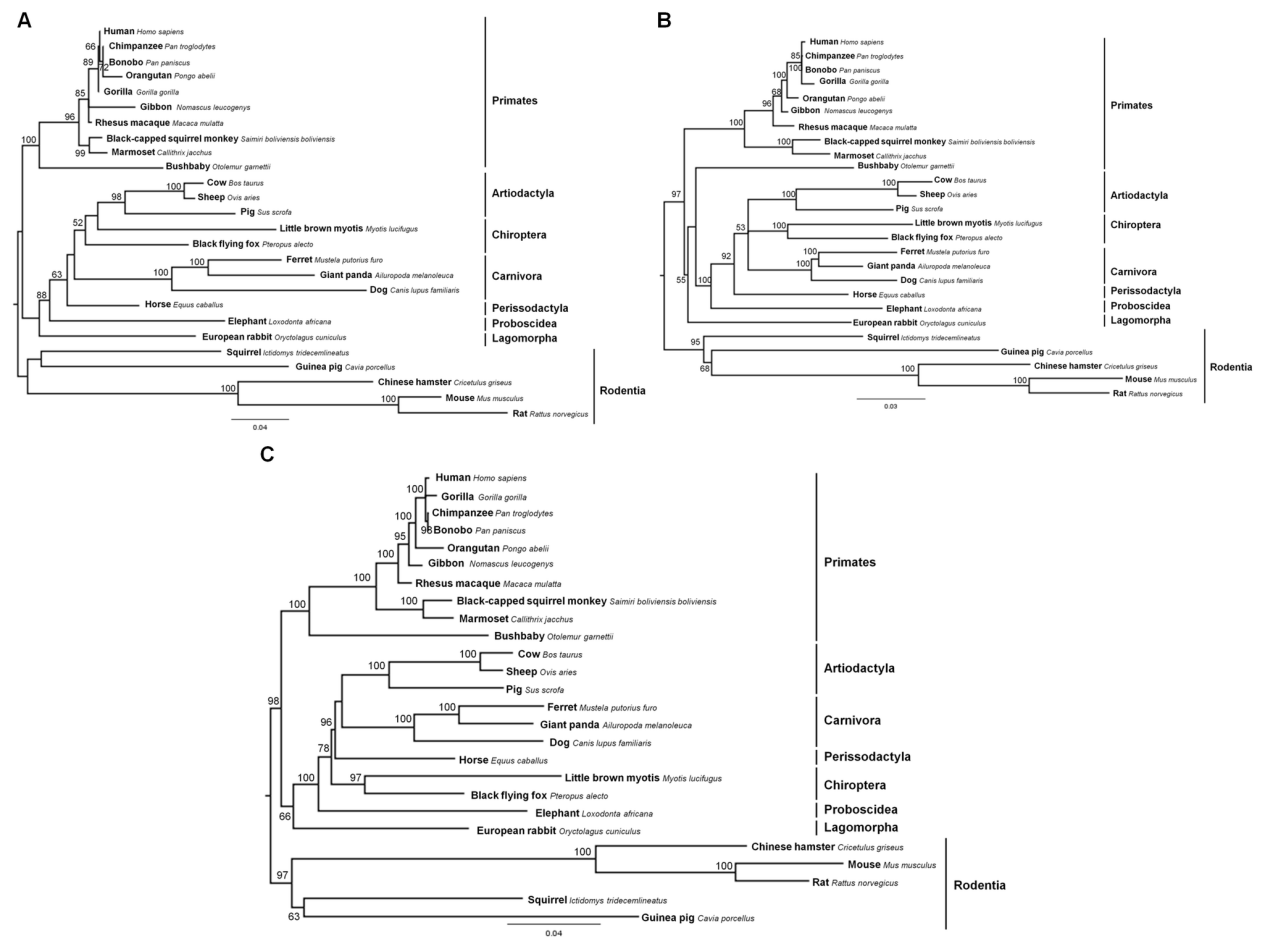
Maximum likelihood (ML) phylogenetic trees of mammalian MDA5 gene used for codon-based ML analysis. When testing mammalian MDA5 alignment for recombination, one significant breakpoint was detected at nucleotide position 903. (A) A phylogenetic tree was reconstructed for the first 903 nucleotides under the nucleotide substitution model TIM3+G. (B) A second ML tree was inferred for the remaining 2211 nucleotides and under the nucleotide substitution model TIM3+I+G. (C) A tree was also reconstructed for MDA5 total alignment without recombination testing and under the nucleotide substitution model GTR+G. Bootstrap values >50 are indicated on the branches.

For the mammalian MDA5 gene sequences alignment, a significant recombination breakpoint was detected (nucleotide 903; p<0.01). Therefore, two ML trees were reconstructed for the resulting segments, one for the first 903 nucleotides (Figure 2A) and another ML tree for the remaining 2211 nucleotides (Figure 2B). The gene phylogeny was also reconstructed for the whole alignment without testing recombination (Figure 2C) to compare its topology with the other two resulting trees. The monophyly of the eight taxonomic orders included in the MDA5 alignment was roughly recovered, with the clear exception of Chiroptera in Figure 2A and Primates in Figure 2B. 

**Figure 3:**
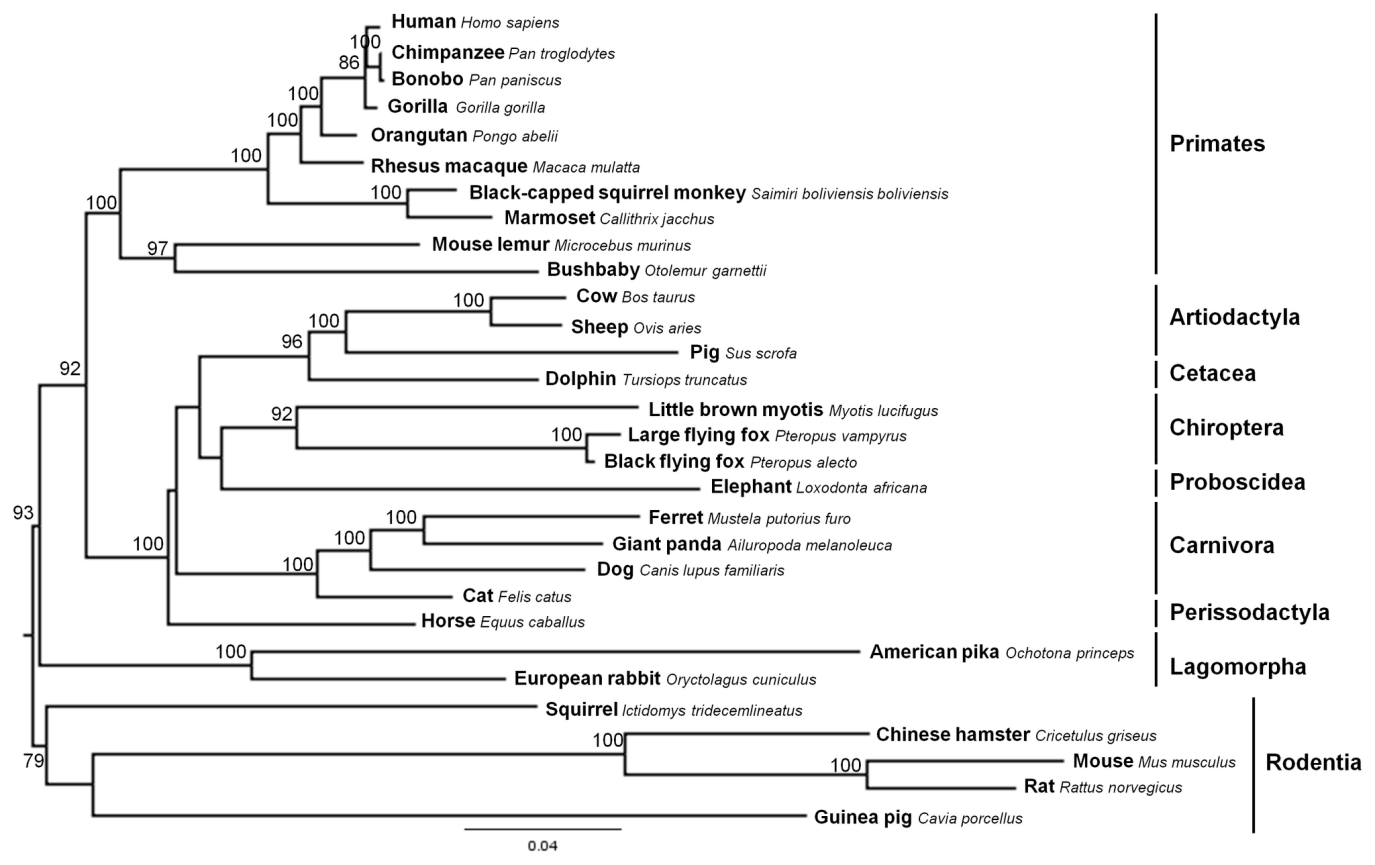
Maximum likelihood (ML) phylogenetic tree of mammalian LGP2 gene used for codon-based ML. The TPM2uf+I+G nucleotide substitution model was employed in mammalian LGP2 gene phylogenetic reconstruction. Bootstrap values >50 are indicated on the branches.

Regarding the LGP2 gene, no clear evidence of recombination was detected. The ML tree obtained (Figure 3) supported the monophyly of the nine mammalian orders collected for this gene.

### Evidence of positive selection in the mammalian RIG-I-like receptors

All the molecular evolutionary analyses in this study were performed for both the complete nucleotide alignments (Figure S1, Figure S2 and Figure S3) and for a trimmed version of the same genes to remove alignment gaps. Figure S7 (RIG-I alignment), Figure S8 (MDA5 alignment) and Figure S9 (LGP2 alignment) correspond to the alignments where gaps present in all sequences, with the exception of one or two, have been removed, while gaps present in only one or two sequences were kept. We observed no significant differences in the results when using one or the other alignment for each gene (data not shown), but ultimately only the results from the trimmed version are presented here.

Evidence for positive selection on mammalian orthologous for RIG-I (Figure S7), MDA5 (Figure S8) and LGP2 (Figure S9) genes was detected using PAML package [54,55] site-specific models M1a versus M2a and M7 versus M8. These models test at the codon level whether a hypothesis that allows for positive selection (models M2a and M8) is a better fit to the data when compared to a null neutral hypothesis (models M1a and M7). Results on the likelihood ratio test (LRT) performed between the likelihood scores of the null neutral and alternative selection models for each gene is indicated in Table 1. Models which allow for positive selection (M2a and M8) gave a significantly better fit to the data for both RIG-I and LGP2 alignments, suggesting that at least some of the codons within each set of orthologous gene sequences are subject to positive selection [56]. Since a recombination breakpoint was detected on the MDA5 alignment, each resulting segment (identified as 1^st^ and 2^nd^ segments) was tested individually for PAML package [54,55] site-specific models. Although the comparison between the null neutral site model M1a and the selection site model M2a did not allow for rejection of the null hypothesis of neutral selection, the comparison between the more powerful pair of site-specific models M7 (neutral) and M8 (selection) yielded significant LRTs (Table 1). 

**Table 1:** RIG-I-like receptors likelihood ratio tests (LRTs) for PAML M1a, M2a, M7 and M8 site models.

PAML site models	lnL_null_^a^	lnL_alternative_^a^	2ΔlnL**^*b*^**	*p*-Value	Tree length
**RIG-I**					4.60
M1a _(nearly neutral)_ vs. M2a _(selection)_	-21065.76	-21045.84	39.84	p<0.0001	
M7 _(neutral, beta)_ vs. M8 _(selection, beta & ω)_	-21053.14	-21016.64	73.00	p<0.0001	
**MDA5_1stSegment**					6.03
M1a _(nearly neutral)_ vs. M2a _(selection)_	-7981.98	-7981.98	0.00	n.s.	
M7 _(neutral, beta)_ vs. M8 _(selection, beta & ω)_	-7970.70	-7966.35	8.70	P<0.02	
**MDA5_2ndSegment**					3.80
M1a _(nearly neutral)_ vs. M2a _(selection)_	-14314.15	-14312.61	3.08	n.s.	
M7 _(neutral, beta)_ vs. M8 _(selection, beta & ω)_	-14284.53	-14272.94	23.18	p<0.0001	
**LGP2**					6.38
M1a _(nearly neutral)_ vs. M2a _(selection)_	-18838.31	-18830.88	14.86	p<0.001	
M7 _(neutral, beta)_ vs. M8 _(selection, beta & ω)_	-18693.92	-18674.22	39.40	p<0.0001	

^a^ lnL: log-likelihood scores.

^b^ 2ΔlnL: likelihood ratio test (LRT) to detect positive selection.

n.s. – non-significant

The PARRIS method [57] implemented in the Datamonkey web server [52,53] was also applied to each RLR trimmed gene alignment (Figure S7, Figure S8 and Figure S9) to look for evidence of positive selection, but no selective pressures were detected in any of the three genes (Table S2).

For each orthologous gene sequences alignment, the tree length parameter is indicated in Table 1. Higher values of tree length, i.e. the expected number of nucleotide substitutions per codon, correspond to higher sequence divergence [58,59]. The tree length values registered for the three genes fell into an intermediate and realistic level of sequence divergence which confers power to the codon models indicated by the LRT scores and to the Bayes empirical Bayes (BEB) approach for site-specific inference of positive selection [58,60].

**Figure 4:**
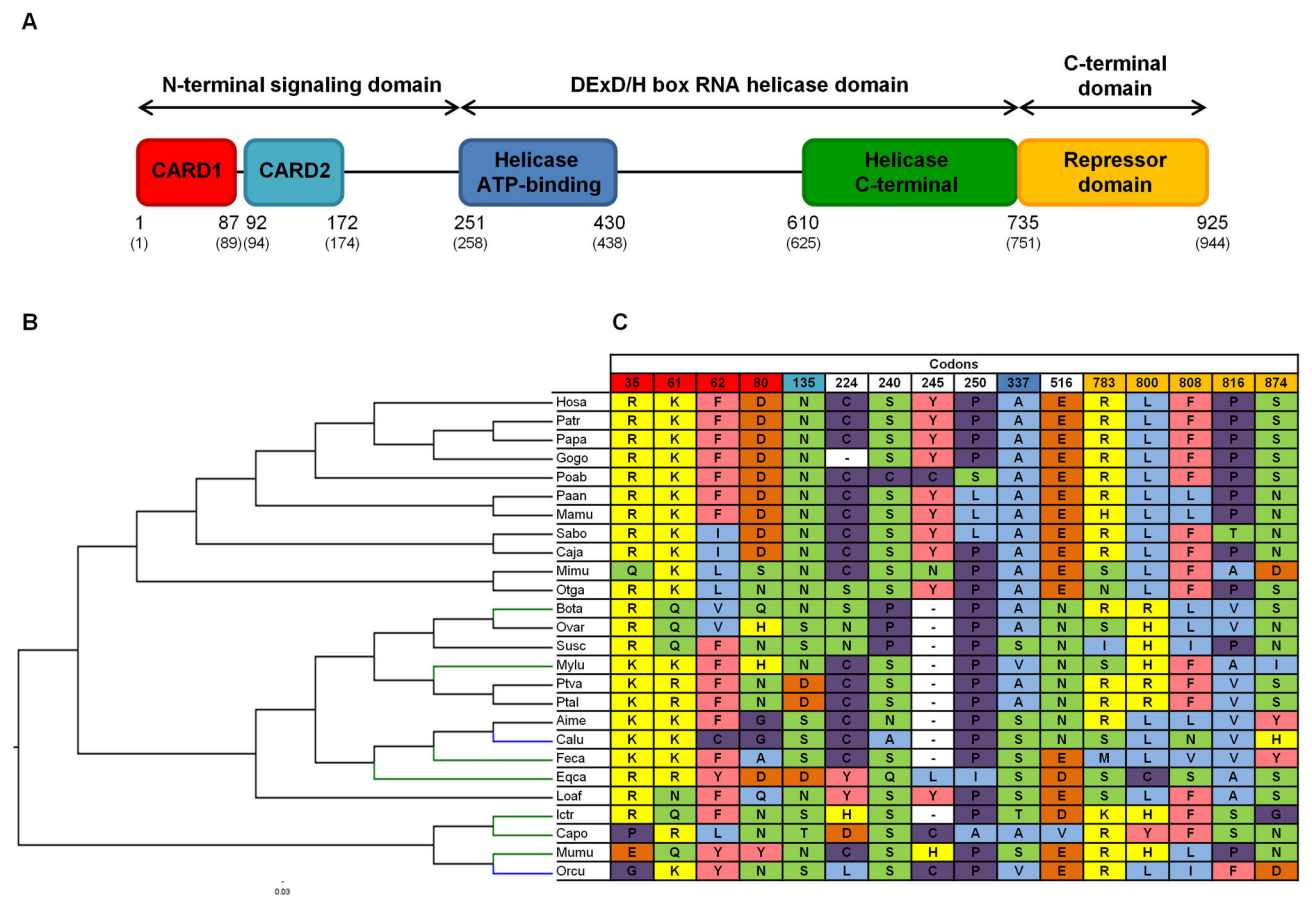
Structural representation and identification of positively-selected branches and codons in mammalian RIG-I. (A) Based on human protein structure, the key domains of RIG-I (http://www.uniprot.org/uniprot/O95786) and the corresponding boundaries are schematically represented. Also, the human domain boundaries while in the mammalian RIG-I deduced protein sequences alignment (Figure S4) are shown in brackets. (B) Cladogram of 26 mammalian RIG-I genes collected from Ensembl and NCBI databases. Branch-site analyses were performed to identify specific branches under episodic positive selection. Branches with statistically significant likelihood ratio tests (LRTs) when performing PAML branch-site model A (Table 2) are colored in green; branches simultaneously identified by PAML branch-site model A and Hyphy branch-site REL method (Table 5) are colored in blue. (C) Positively-selected codons are exhibited in the table and numbered according to the mammalian RIG-I deduced protein sequences alignment (Figure S4). Symbol “-” represents a deletion. Colors on the codon numbering row correspond to the RIG-I domain with the same color in the protein structural representation (A). The background colors on the identified sites match different amino acid properties: polar positive (yellow), polar negative (orange), polar neutral (green), non-polar neutral (purple), non-polar aliphatic (blue) and non-polar aromatic (pink). The used abbreviations correspond, by order of appearance, to the following species: Hosa – Human; Patr – Chimpanzee; Papa – Bonobo; Gogo – Gorilla; Poab – Orangutan; Paan – Olive baboon; Mamu – Rhesus macaque; Sabo – Black-capped squirrel monkey; Caja – Marmoset; Mimu – Mouse lemur; Otga – Bushbaby; Bota – Cow; Ovar – Sheep; Susc – Pig; Mylu – Little brown myotis; Ptva – Large flying fox; Ptal – Black flying fox; Aime – Giant panda; Calu – Dog; Feca – Cat; Eqca – Horse; Loaf – Elephant; Ictr – Squirrel; Capo – Guinea pig; Mumu – Mouse; Orcu – European rabbit.

**Figure 5:**
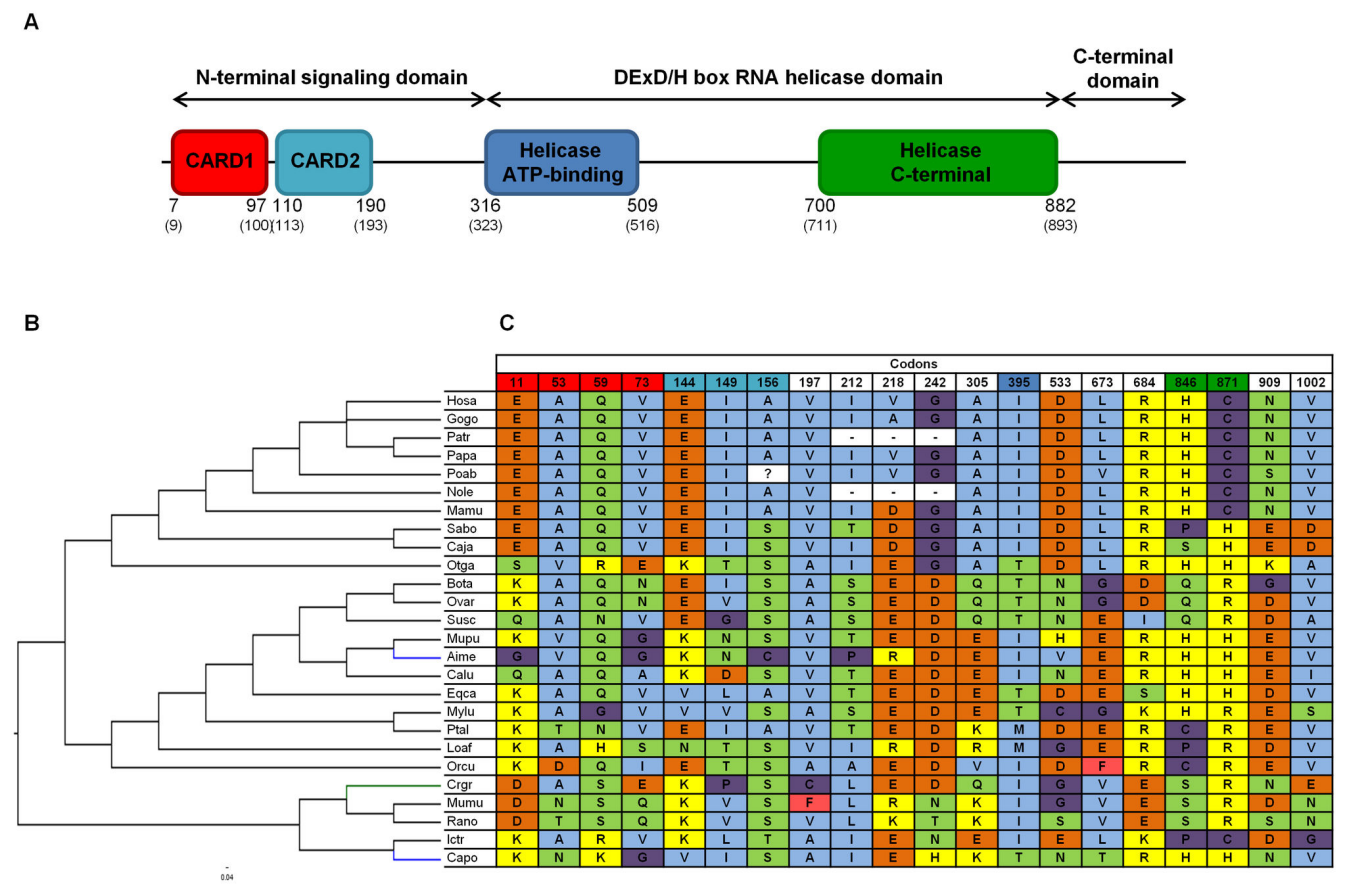
Structural representation and identification of positively-selected branches and codons in mammalian MDA5. (A) Based on the human protein structure, the key domains of MDA5 (http://www.uniprot.org/uniprot/Q9BYX4) and the corresponding boundaries are schematically represented. Also, the human domain boundaries while in the mammalian MDA5 deduced protein sequences alignment (Figure S5) are shown in brackets. (B) Cladogram of 26 mammalian MDA5 genes collected from Ensembl and NCBI databases. Branch-site analyses were performed to identify specific branches episodic under positive selection. Branches with statistically significant likelihood ratio tests (LRTs) when performing PAML branch-site model A (Table 3) are colored in green; branches simultaneously identified by PAML branch-site model A and Hyphy branch-site REL method (Table 5) are colored in blue. (C) Positively-selected codons are exhibited in the table and numbered according to the mammalian MDA5 deduced protein sequences alignment (Figure S5). Symbol “?” represents an undetermined amino acid, while “-” symbolizes a deletion. Colors on the codon numbering row correspond to the MDA5 domain with the same color in the protein structural representation (A). The background colors on the identified sites match different amino acid properties: polar positive (yellow), polar negative (orange), polar neutral (green), non-polar neutral (purple), non-polar aliphatic (blue) and non-polar aromatic (pink). The used abbreviations correspond, by order of appearance, to the following species: Hosa – Human; Gogo – Gorilla; Patr – Chimpanzee; Papa – Bonobo; Poab – Orangutan; Nole – Gibbon; Mamu – Rhesus macaque; Sabo – Black-capped squirrel monkey; Caja – Marmoset; Otga – Bushbaby; Bota – Cow; Ovar – Sheep; Susc – Pig; Mupu – Ferret; Aime – Giant panda; Calu – Dog; Eqca – Horse; Mylu – Little brown myotis; Ptal – Black flying fox; Loaf – Elephant; Orcu – European rabbit; Crgr – Chinese hamster; Mumu – Mouse; Rano – Rat; Ictr – Squirrel; Capo – Guinea pig.

**Figure 6:**
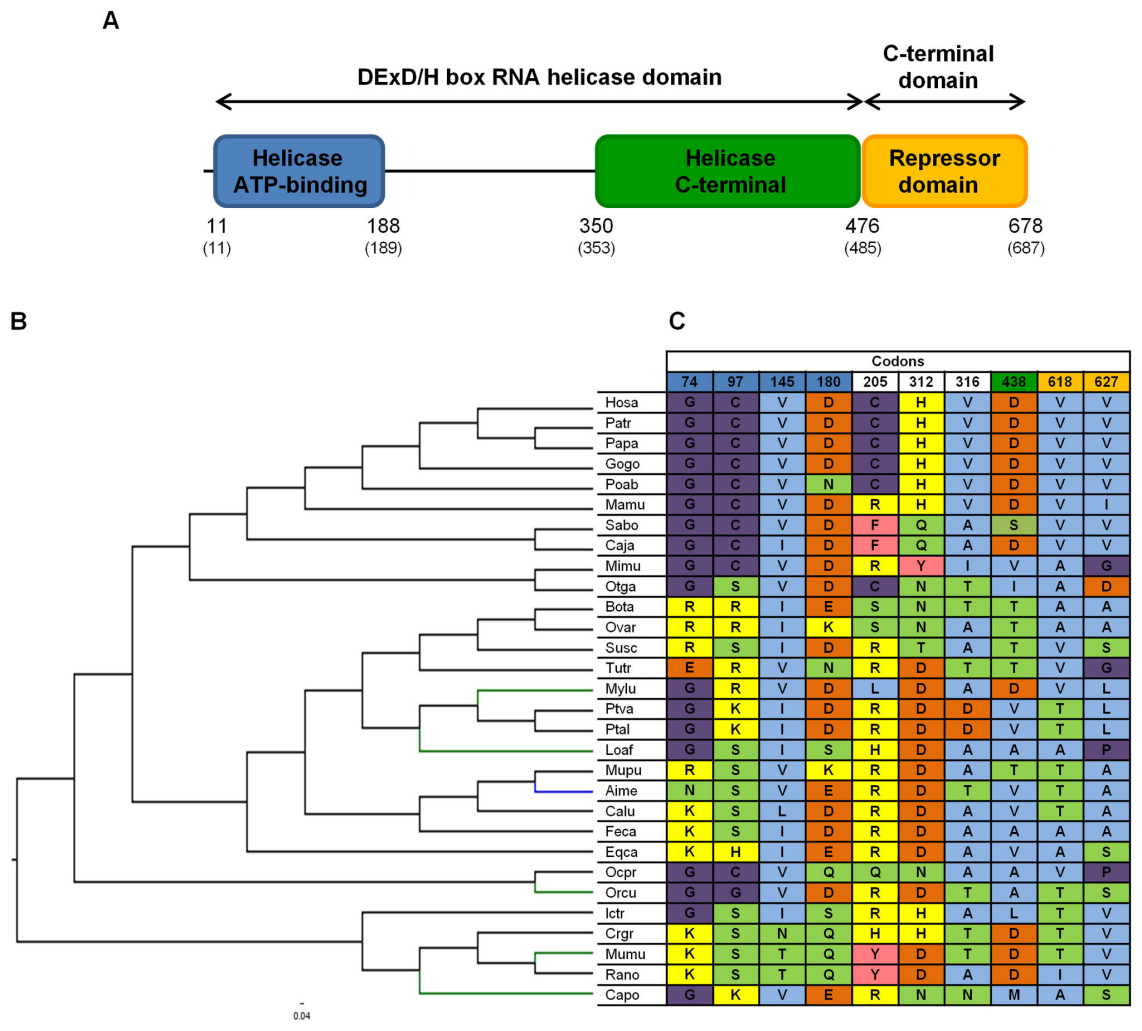
Structural representation and identification of positively-selected branches and codons in mammalian LGP2. (A) Based on human protein structure, the key domains of LGP2 (http://www.uniprot.org/uniprot/Q96C10) and the corresponding boundaries are schematically represented. Also, the human domain boundaries while in the mammalian LGP2 deduced protein sequences alignment (Figure S6) are shown in brackets. (B) Cladogram of 30 mammalian LGP2 genes collected from Ensembl and NCBI databases. Branch-site analyses were performed to identify specific branches under episodic positive selection. Branches with statistically significant likelihood ratio tests (LRTs) when performing PAML branch-site model A (Table 4) are colored in green; branch colored in blue has been simultaneously identified by PAML branch-site model A and Hyphy branch-site REL method (Table 5). (C) Positively-selected codons are exhibited in the table and numbered according to the mammalian LGP2 deduced protein sequences alignment (Figure S6). Colors on the codon numbering row correspond to the LGP2 domain with the same color in the protein structural representation (A). The background colors on the identified codons match different amino acid properties: polar positive (yellow), polar negative (orange), polar neutral (green), non-polar neutral (purple), non-polar aliphatic (blue) and non-polar aromatic (pink). The used abbreviations correspond, by order of appearance, to the following species: Hosa – Human; Patr – Chimpanzee; Papa – Bonobo; Gogo – Gorilla; Poab – Orangutan; Mamu – Rhesus macaque; Sabo – Black-capped squirrel monkey; Caja – Marmoset; Mimu – Mouse lemur; Otga – Bushbaby; Bota – Cow; Ovar – Sheep; Susc – Pig; Tutr – Dolphin; Mylu – Little brown myotis; Ptva – Large flying fox; Ptal – Black flying fox; Loaf – Elephant; Mupu – Ferret; Aime – Giant panda; Calu – Dog; Feca – Cat; Eqca – Horse; Ocpr – American pika; Orcu – European rabbit; Ictr – Squirrel; Crgr – Chinese hamster; Mumu – Mouse; Rano – Rat; Capo – Guinea pig.

Model M8 implemented in the PAML package [54,55] and Datamonkey web server [52,53] SLAC, FEL, REL, MEME and FUBAR methods [61-63] were used to detect specific codons under selection in the three RLR genes. Based on the methodology adopted by other authors and in previous studies [43,47,48,64], only codons identified by at least three of the six used methods are considered to be under positive selection (Table S3). Since the breadth of species included in each alignment is wide, by applying several methods to detect codons under positive selection and by overlapping the results, we should be decreasing the incidence of false positives. A total of sixteen codons for RIG-I (Figure 4), twenty for MDA5 (Figure 5) and ten for LGP2 (Figure 6) were identified as candidate codons under selective pressure. Regarding their location in each corresponding protein, the greatest number of these codons are located in protein functional domains, more specifically, eleven out of the sixteen RIG-I codons (~ 69%), ten out of the twenty MDA5 codons (50%) and seven out of the ten LGP2 codons (70%). To estimate the percentage of positively selected codons in the three proteins, we used human deduced protein sequences as a reference. Human LGP2 exhibited 1.47% (10/678) of codons under positive selection. Higher values were obtained for human MDA5 and RIG-I, 1.95% (20/1025) and 1.73% (16/925) of codons under selective pressure, respectively.

To detect signatures of episodic positive selection in specific lineages of each RLR orthologous gene sequences alignment we performed two branch-site model analyses. These models allow the selective pressure indicated by the nonsynonymous to synonymous substitution rate ratio ω (d_N_/d_S_) to vary both across sites in the gene and across lineages on the tree [65]. Since no biological hypothesis existed to specify *a priori* branches to be examined for positive selection, the branch-site model A implemented in the PAML package [54,55,66] was applied to all species branches on each RLR gene phylogenetic tree. The LRT performed for each branch was significant for 2ΔlnL > 3.84 [55,66]. Our analyses suggest that nine species branches in RIG-I are under selective pressure (Table 2 and Figure 4B). Branch-site model A was applied to the two MDA5 trees resultant from recombination testing and, for each tree, positive selection has operated only in two species branches (Table 3 and Figure 5B). For LGP2, a total of six species branches had significant LRTs corresponding to candidate lineages under selection (Table 4 and Figure 6B). Some of the species branches recognized by the branch-site model A were also identified by the branch-site REL method [67] (Table 5) available in the Datamonkey web server [52,53]. For both RIG-I and MDA5, two species branches were simultaneously identified by the two methods, consisting in dog (Calu) and European rabbit (Orcu) branches for RIG-I (Figure 4B) and giant panda (Aime) and Guinea pig (Capo) branches for MDA5 (Figure 5B). Only one LGP2 species branch, corresponding to the giant panda (Aime), was simultaneously identified by the branch-site model A and the branch-site REL method (Figure 6B). 

**Table 2:** PAML branch-site model A analysis to identify branches under episodic positive selection in RIG-I phylogenetic tree.

Foreground branches**^a^**	Parameters under null model	lnL^b^ (null)	Parameters under alternative model	lnL^b^ (alternative)	2ΔlnL**^c^**	*p*-Value	Positively selected sites**^d^**
Bota	*p*_*0*_ = 0.536 *p*_*1*_ = 0.294 *p*_*2a*_ = 0.110 *p*_*2b*_ = 0.060 *ω*_*0*_ = 0.090 *ω*_*1*_ = 1 *ω*_*2*_ = 1	-21065.614	*p*_*0*_ = 0.637 *p*_*1*_ = 0.349 *p*_*2a*_ = 0.009 *p*_*2b*_ = 0.005 *ω*_*0*_ = 0.090 *ω*_*1*_ = 1 *ω*_*2*_ = **18.749**	-21063.247	**4.734**	<0.05	**655**F (0.907) **656**Q (0.996)
Calu	*p*_*0*_ = 0.631 *p*_*1*_ = 0.343 *p*_*2a*_ = 0.016 *p*_*2b*_ = 0.009 *ω*_*0*_ = 0.092 *ω*_*1*_ = 1 *ω*_*2*_ = 1	-21069.596	*p*_*0*_ = 0.645 *p*_*1*_ = 0.350 *p*_*2a*_ = 0.003 *p*_*2b*_ = 0.002 *ω*_*0*_ = 0.093 *ω*_*1*_ = 1 *ω*_*2*_ = **70.492**	-21065.810	**7.572**	<0.01	none
Capo	*p*_*0*_ = 0.617 *p*_*1*_ = 0.335 *p*_*2a*_ = 0.031 *p*_*2b*_ = 0.017 *ω*_*0*_ = 0.090 *ω*_*1*_ = 1 *ω*_*2*_ = 1	-21068.600	*p*_*0*_ = 0.641 *p*_*1*_ = 0.347 *p*_*2a*_ = 0.008 *p*_*2b*_ = 0.004 *ω*_*0*_ = 0.092 *ω*_*1*_ = 1 *ω*_*2*_ = **^*e*^**	-21065.132	**6.936**	<0.01	none
Eqca	*p*_*0*_ = 0.648 *p*_*1*_ = 0.352 *p*_*2a*_ = 0 *p*_*2b*_ = 0 *ω*_*0*_ = 0.093 *ω*_*1*_ = 1 *ω*_*2*_ = 1	-21069.711	*p*_*0*_ = 0.643 *p*_*1*_ = 0.351 *p*_*2a*_ = 0.004 *p*_*2b*_ = 0.002 *ω*_*0*_ = 0.092 *ω*_*1*_ = 1 *ω*_*2*_ = **47.106**	-21067.540	**4.342**	<0.05	none
Feca	*p*_*0*_ = 0.613 *p*_*1*_ = 0.333 *p*_*2a*_ = 0.035 *p*_*2b*_ = 0.019 *ω*_*0*_ = 0.092 *ω*_*1*_ = 1 *ω*_*2*_ = 1	-21068.674	*p*_*0*_ = 0.643 *p*_*1*_ = 0.351 *p*_*2a*_ = 0.004 *p*_*2b*_ = 0.002 *ω*_*0*_ = 0.092 *ω*_*1*_ = 1 *ω*_*2*_ = **265.180**	-21064.248	**8.852**	<0.005	**44**W (0.943)
Ictr	*p*_*0*_ = 0.611 *p*_*1*_ = 0.331 *p*_*2a*_ = 0.038 *p*_*2b*_ = 0.020 *ω*_*0*_ = 0.091 *ω*_*1*_ = 1 *ω*_*2*_ = 1	-21068.446	*p*_*0*_ = 0.644 *p*_*1*_ = 0.348 *p*_*2a*_ = 0.005 *p*_*2b*_ = 0.003 *ω*_*0*_ = 0.093 *ω*_*1*_ = 1 *ω*_*2*_ = **16.527**	-21066.281	**4.330**	<0.05	none
Mumu	*p*_*0*_ = 0.648 *p*_*1*_ = 0.352 *p*_*2a*_ = 0 *p*_*2b*_ = 0 *ω*_*0*_ = 0.093 *ω*_*1*_ = 1 *ω*_*2*_ = 1	-21069.711	*p*_*0*_ = 0.646 *p*_*1*_ = 0.346 *p*_*2a*_ = 0.005 *p*_*2b*_ = 0.003 *ω*_*0*_ = 0.093 *ω*_*1*_ = 1 *ω*_*2*_ = **^*f*^**	-21067.171	**5.080**	<0.025	none
Mylu	*p*_*0*_ = 0.648 *p*_*1*_ = 0.352 *p*_*2a*_ = 0 *p*_*2b*_ = 0 *ω*_*0*_ = 0.093 *ω*_*1*_ = 1 *ω*_*2*_ = 1	-21069.711	*p*_*0*_ = 0.645 *p*_*1*_ = 0.351 *p*_*2a*_ = 0.002 *p*_*2b*_ = 0.001 *ω*_*0*_ = 0.093 *ω*_*1*_ = 1 *ω*_*2*_ = **172.548**	-21066.025	**7.372**	<0.01	none
Orcu	*p*_*0*_ = 0.599 *p*_*1*_ = 0.320 *p*_*2a*_ = 0.053 *p*_*2b*_ = 0.028 *ω*_*0*_ = 0.089 *ω*_*1*_ = 1 *ω*_*2*_ = 1	-21064.127	*p*_*0*_ = 0.637 *p*_*1*_ = 0.336 *p*_*2a*_ = 0.017 *p*_*2b*_ = 0.009 *ω*_*0*_ = 0.092 *ω*_*1*_ = 1 *ω*_*2*_ = **38.747**	-21046.263	**35.728**	<0.001	**849**T (0.989) **851**C (0.997) **854**S (0.958) **857H** (0.988) **861**G (0.982) **898**V (0.989)

^a^ Species names on the foreground branches: Bota – Cow; Calu – Dog; Capo – Guinea pig; Eqca – Horse; Feca – Cat; Ictr – Squirrel; Mumu – Mouse; Mylu – Little brown myotis; Orcu – European rabbit.

^b^ lnL: log-likelihood scores.

^c^ 2ΔlnL: likelihood ratio test (LRT) to detect positive selection.

^d^ Positively selected sites: posterior probabilities >0.90 in the BEB (Bayes Empirical Bayes) analyses.

^e^ *ω*_*2*_ parameter varied for different (*ω*) values: *ω*_*2*_ (2) = 242.957; *ω*_*2*_ (3) = 340.801; *ω*_*2*_ (4) = 982.380.

^f^ *ω*_*2*_ parameter varied for different (*ω*) values: *ω*_*2*_ (2) = 131.879; *ω*_*2*_ (3) = 246.814; *ω*_*2*_ (4) = 289.634.

**Table 3:** PAML branch-site model A analysis to identify branches under episodic positive selection in MDA5 phylogenetic trees.

Foreground branches **^a^**	Parameters under null model	lnL^b^ (null)	Parameters under alternative model	lnL^b^ (alternative)	2ΔlnL**^c^**	*p*-Value	Positively selected sites**^d^**
**1^st^ Segment**							
Aime	*p*_*0*_ = 0.506 *p*_*1*_ = 0.377 *p*_*2a*_ = 0.067 *p*_*2b*_ = 0.050 *ω*_*0*_ = 0.086 *ω*_*1*_ = 1 *ω*_*2*_ = 1	-7981.227	*p*_*0*_ = 0.559 *p*_*1*_ = 0.418 *p*_*2a*_ = 0.013 *p*_*2b*_ = 0.010 *ω*_*0*_ = 0.086 *ω*_*1*_ = 1 *ω*_*2*_ = **27.801**	-7976.648	**9.158**	<0.005	**295**P (0.995)
Capo	*p*_*0*_ = 0.488 *p*_*1*_ = 0.364 *p*_*2a*_ = 0.085 *p*_*2b*_ = 0.063 *ω*_*0*_ = 0.077 *ω*_*1*_ = 1 *ω*_*2*_ = 1	-7973.674	*p*_*0*_ = 0.543 *p*_*1*_ = 0.409 *p*_*2a*_ = 0.027 *p*_*2b*_ = 0.021 *ω*_*0*_ = 0.078 *ω*_*1*_ = 1 *ω*_*2*_ = **27.756**	-7964.534	**18.280**	<0.001	**281K** (0.972) **284**F (0.993) **291**P (0.996) **293L** (0.989) **297**I (0.998)
**2^nd^ Segment**							
Capo	*p*_*0*_ = 0.727 *p*_*1*_ = 0.205 *p*_*2a*_ = 0.053 *p*_*2b*_ = 0.015 *ω*_*0*_ = 0.070 *ω*_*1*_ = 1 *ω*_*2*_ = 1	-14272.950	*p*_*0*_ = 0.759 *p*_*1*_ = 0.209 *p*_*2a*_ = 0.025 *p*_*2b*_ = 0.007 *ω*_*0*_ = 0.073 *ω*_*1*_ = 1 *ω*_*2*_ = **998.992**	-14255.172	**35.556**	<0.001	**358**G (0.958) **593**S (1) **594**S (1) **595L** (0.990)
Crgr	*p*_*0*_ = 0.778 *p*_*1*_ = 0.222 *p*_*2a*_ = 0 *p*_*2b*_ = 0 *ω*_*0*_ = 0.074 *ω*_*1*_ = 1 *ω*_*2*_ = 1	-14281.640	*p*_*0*_ = 0.778 *p*_*1*_ = 0.219 *p*_*2a*_ = 0.003 *p*_*2b*_ = 0.001 *ω*_*0*_ = 0.075 *ω*_*1*_ = 1 *ω*_*2*_ = **^*e*^**	-14277.837	**7.606**	<0.01	none

^a^ Species names on the foreground branches: Aime – Giant panda; Capo – Guinea pig; Crgr – Chinese hamster.

^b^ lnL: log-likelihood scores.

^c^ 2ΔlnL: likelihood ratio test (LRT) to detect positive selection.

^d^ Positively selected sites: posterior probabilities >0.90 in the BEB (Bayes Empirical Bayes) analyses.

^e^ *ω*_*2*_ parameter varied for different (*ω*) values: *ω*_*2*_ (2) = 999.000; *ω*_*2*_ (3) = 832.570; *ω*_*2*_ (4) = 681.973.

**Table 4:** PAML branch-site model A analysis to identify branches under episodic positive selection in LGP2 phylogenetic tree.

Foreground branches **^a^**	Parameters under null model	lnL^b^ (null)	Parameters under alternative model	lnL^b^ (alternative)	2ΔlnL**^c^**	*p*-Value	Positively selected sites**^d^**
Aime	*p*_*0*_ = 0.729 *p*_*1*_ = 0.182 *p*_*2a*_ = 0.070 *p*_*2b*_ = 0.018 *ω*_*0*_ = 0.082 *ω*_*1*_ = 1 *ω*_*2*_ = 1	-18816.537	*p*_*0*_ = 0.794 *p*_*1*_ = 0.199 *p*_*2a*_ = 0.005 *p*_*2b*_ = 0.001 *ω*_*0*_ = 0.082 *ω*_*1*_ = 1 *ω*_*2*_ = **86.683**	-18810.272	**12.530**	<0.001	**227L** (0.913) **519**C (0.999) **520N** (0.995)
Capo	*p*_*0*_ = 0.794 *p*_*1*_ = 0.198 *p*_*2a*_ = 0.006 *p*_*2b*_ = 0.002 *ω*_*0*_ = 0.083 *ω*_*1*_ = 1 *ω*_*2*_ = 1	-18821.176	*p*_*0*_ = 0.795 *p*_*1*_ = 0.197 *p*_*2a*_ = 0.006 *p*_*2b*_ = 0.001 *ω*_*0*_ = 0.083 *ω*_*1*_ = 1 *ω*_*2*_ = **12.697**	-18819.037	**4.278**	<0.05	**176N** (0.929) **267**S (0.979)
Loaf	*p*_*0*_ = 0.787 *p*_*1*_ = 0.196 *p*_*2a*_ = 0.014 *p*_*2b*_ = 0.003 *ω*_*0*_ = 0.083 *ω*_*1*_ = 1 *ω*_*2*_ = 1	-18820.429	*p*_*0*_ = 0.792 *p*_*1*_ = 0.196 *p*_*2a*_ = 0.009 *p*_*2b*_ = 0.002 *ω*_*0*_ = 0.083 *ω*_*1*_ = 1 *ω*_*2*_ = **423.742**	-18812.759	**15.340**	<0.001	**421**S (0.996) **569**S (0.985)
Mimu	*p*_*0*_ = 0.801 *p*_*1*_ = 0.199 *p*_*2a*_ = 0 *p*_*2b*_ = 0 *ω*_*0*_ = 0.083 *ω*_*1*_ = 1 *ω*_*2*_ = 1	-18821.530	*p*_*0*_ = 0.797 *p*_*1*_ = 0.198 *p*_*2a*_ = 0.004 *p*_*2b*_ = 0.001 *ω*_*0*_ = 0.083 *ω*_*1*_ = 1 *ω*_*2*_ = **775.269**	-18819.489	**4.082**	<0.05	none
Mylu	*p*_*0*_ = 0.769 *p*_*1*_ = 0.190 *p*_*2a*_ = 0.033 *p*_*2b*_ = 0.008 *ω*_*0*_ = 0.082 *ω*_*1*_ = 1 *ω*_*2*_ = 1	-18819.788	*p*_*0*_ = 0.795 *p*_*1*_ = 0.193 *p*_*2a*_ = 0.009 *p*_*2b*_ = 0.002 *ω*_*0*_ = 0.083 *ω*_*1*_ = 1 *ω*_*2*_ = **11.149**	-18817.547	**4.482**	<0.05	**646**A (0.980)
Orcu	*p*_*0*_ = 0.779 *p*_*1*_ = 0.193 *p*_*2a*_ = 0.022 *p*_*2b*_ = 0.006 *ω*_*0*_ = 0.083 *ω*_*1*_ = 1 *ω*_*2*_ = 1	-18820.688	*p*_*0*_ = 0.797 *p*_*1*_ = 0.197 *p*_*2a*_ = 0.005 *p*_*2b*_ = 0.001 *ω*_*0*_ = 0.083 *ω*_*1*_ = 1 *ω*_*2*_ = **63.328**	-18817.321	**6.734**	<0.01	**605**P (0.961)

^a^ Species names on the foreground branches: Aime – Giant panda; Capo – Guinea pig; Loaf – Elephant; Mimu – Mouse lemur; Mylu – Little brown myotis; Orcu – European rabbit.

^b^ lnL: log-likelihood scores.

^c^ 2ΔlnL: likelihood ratio test (LRT) to detect positive selection.

^d^ Positively selected sites: posterior probabilities >0.90 in the BEB (Bayes Empirical Bayes) analyses.

**Table 5:** Hyphy branch-site REL analysis to identify RIG-I-like receptor species branches subject to episodic diversifying selection.

Branch**^a^**	ω^+ b^	*p*-Value
**RIG-I**		
Calu	57.89	0.032
Orcu	30.23	< 0.0001
**MDA5**		
Aime	11.46	0.004
Capo	3334.49	< 0.0001
**LGP2**		
Aime	196.66	0.043

^a^ Species names on the branches: Aime – Giant panda; Calu – Dog; Capo – Guinea pig; Orcu – European rabbit.

^b^ ω^+^: strength and extent (proportion of sites) of selection along each branch.

## Discussion

In a human population genetics context, the first study on RLRs evolutionary history and selective footprints has been recently published [68]. Nevertheless, to the best of our knowledge, our study is the first that searches for selective pressure acting on mammalian orthologous of the three RLRs and, in fact, we provide strong evidence of positive selection as well as identify a significant number of codons under probable selective pressures for RIG-I, MDA5 and LGP2. Furthermore, our results on the RIG-I RD in specific hosts suggest that certain viruses might be exerting long-term selective pressures on this gene.

TLRs adaptive evolution has been the most extensively characterized of all the PRRs in several animal groups, such as echinoderms [69], birds [70] and different mammals [43,64,71-74]. Studies on known viral-recognition TLRs (TLR3, 7, 8 and 9) of closely related animal groups, like birds [70], or within species, like humans and chimpanzees [64], demonstrated that this class of PRRs exhibits a background of strong purifying selection to keep their functional integrity, albeit in the birds study [70] significant instances of positive selection acting on a few amino acid sites were identified. Nevertheless, when different ML codon-based methods were applied to detect evidence of acting positive selection in broader groups where a great number of species are included, like primates [64] and mammals [43], most of the viral TLRs exhibited strong evidence of positive selection and specific codons with a high probability of being under selection were identified. Similarly, in our study the codon-based analyses strongly support that the three RLR genes, RIG-I, MDA5 and LGP2, have all been subject to long-term selective pressures during mammalian evolution. Also, we applied several methods that identified specific RLR codons with a high probability of being under selection, which may directly perturb downstream immune responses in a particular host infected by a viral pathogen. 

One of the major concerns when using large scale divergent species to infer positive selection acting on a set of orthologous genes and across lineages on the phylogenetic tree is the effect of saturation in synonymous substitutions, since they may saturate quickly as sequences diverge [75,76]. As codon models consider both synonymous and nonsynonymous substitutions, the saturation of the first could cloud the information provided by nonsynonymous substitutions. Nevertheless, the sequence divergence in our study, inferred through RLRs tree length values, fit into intermediate and realistic levels that should confer power to the LRT used to compare nested codon-models and robustness to the branch-site models, and to the BEB approach for codon-specific detection of positive selection [58-60]. Also, in this study the mammalian species collected for each of the three RLR genes were nearly the same, thus this host species spectrum should not influence the codon-based analyses and our observations when comparing the level of selective pressure between genes.

In our study, mammalian MDA5 showed the highest number and percentage of positively selected codons. Nonetheless, the percentage of MDA5 codons under selection located in the known protein functional domains was the lowest. This should reflect the imposition of functional and structural constraints in MDA5 defined domains. On the other hand, we observed that LGP2 is apparently less prone to evolutionary change with the lowest number and percentage of codons under selective pressures. For RIG-I, the greatest number of codons identified as candidates under selective pressures were located in known protein functional domains, which might reveal the pressure imposed by the great number of viruses recognized by this RLR [13,14]. Vasseur and colleagues [68] came to different conclusions in their study, once they were focused on intra-species (human populations) polymorphisms and on the comparison of nonsynonymous to synonymous rates ratio ω (d_N_/d_S_) between human and chimpanzee lineages for the three RLR genes. RIG-I exhibited a stronger evolutionary constraint [68], as attested by its low levels of nucleotide diversity, population differentiation and low tolerance of amino acid-altering variation. It also exhibited a dramatic decay in the ω ratio when compared to the other two RLRs [68]. This is the expected outcome in evolutionary studies when using closely related species, or genetic information for population of the same species, which result in a background of strong purifying selection to keep the protein functional integrity. In the same study [68], the strongest signatures of positive selection were found in MDA5 and LGP2 by exhibiting higher ω ratios than RIG-I. Besides, MDA5 and LGP2 also appear to have evolved adaptively in specific human populations, presenting a great number of nonsynonymous mutations in both helicase and C-terminal domains [68].

RIG-I and MDA5 contain two N-terminal CARDs [10,17]. The interaction of these domains with an adaptor protein named IPS-1 (also known as MAVS, VISA or CARDIF) is a crucial process to activate a wide range of downstream response factors, including type I IFNs and other essential anti-viral proteins to induce intracellular immune responses [77]. Interestingly, in our study, the CARDs of both RIG-I and MDA5 concentrated a large number of the deduced codons under selection. Some of these are radical in terms of their physicochemical properties changes across mammalian species (Figure 4 and Figure 5), strengthening the case for positive selection. Since the two CARDs are fundamental for downstream RIG-I and MDA5 signaling, which implies functional constraints, the observed variability across species can be perceived as a great structural plasticity for mammalian CARDs.

The helicase domain in the RLR family is generally described as exhibiting affinity for dsRNA [78]. The existence of six highly conserved sequence motifs within this domain is a characteristic of the helicase superfamily 2 which integrates DExD/H box RNA helicases. Also, different aspects of helicase functions have been assigned to specific motifs [79]. Bamming and Horvath [11] compared the amino acid sequences of the three human RLR helicase domains with the established consensus sequences of helicase families elements and, despite slight differences, the sequences in individual motifs are highly conserved within RIG-I, MDA5 and LGP2. Indeed, in our study the six helicase motifs of the three proteins were evolutionary conserved (data not shown) in the mammalian species collected. Minor alterations occur in some species, but the extent of those differences concerning the involvement in substrate interaction, signal transduction and/or the whole anti-viral response profile, is difficult to predict. 

RIG-I RD is responsible for recognizing and binding to its RNA substrates in a 5’-triphosphate (5’-ppp)-dependent manner. Besides, binding studies clearly established that the pppRNA binding site resides within the RD [14,26,80]. The function described for RIG-I RD makes our current results worthy of note, since the RD is the RIG-I domain that exhibits the strongest evidence of trans-acting selective pressures (Figure 4). Whether these differences play a role in RIG-I activation after binding to the RNAs from different viral pathogens that infect distinct mammalian hosts is a complex question. Nevertheless, we can assume that the RD variability in mammals is related to the fact that RIG-I senses a large variety of viruses [13,14].

The performance of branch-site models in our study imposes a careful interpretation of data, since only one representative element of each species was included. Still, some branches of the three RLR phylogenetic trees exhibited evidence of positive selection. The two species under episodic positive selection on RIG-I phylogenetic tree, the domestic dog and the European rabbit, are susceptible hosts of two viruses recognized by RIG-I, rabies virus (Rhabdoviridae family) and myxoma virus (Poxviridae family), respectively [23,39]. Such results suggest that these lethal pathogens, and possibly other re-occurring viral infections in these specific hosts, might be exerting long-term selective pressures on the susceptible host RIG-I gene. Therefore, the changes on RIG-I sequences across species, with special focus on the RD as suggested above, should be the result of a co-evolutionary process between species-specific infecting viruses and this host RNA sensor protein.

By detecting the extension of acting positive selection on mammalian RLRs, this study provides further insights into their biological functions in host defense against viral pathogens in general. Differences in these genes across mammalian species may consequently impact downstream immune responses and, as a result, contribute to the species-specific resistance/susceptibility profiles against many diverse viral pathogens. 

## Materials and Methods

### Mammalian RIG-I-like receptor gene sequences

The coding region of the three RLR genes, RIG-I, MDA5 and LGP2, were collected for different mammalian species from NCBI (http://www.ncbi.nlm.nih.gov) and Ensembl (http://www.ensembl.org/index.html) databases (Table S1). Each set of mammalian orthologous gene sequences was aligned with ClustalW [81] implemented in BioEdit v7.0.9 [82]. The nucleotide sequences alignment corresponding to each gene coding region is represented in Figure S1 (RIG-I alignment), Figure S2 (MDA5 alignment) and Figure S3 (LGP2 alignment). Translation into protein sequences was performed using also BioEdit [82]. Figure S4, Figure S5 and Figure S6 represent the alignments of the deduced protein sequences for RIG-I, MDA5 and LGP2, respectively. For the evolutionary analyses, representative alignment gaps in Figure S1, Figure S2 and Figure S3 had to be removed: gaps present in all sequences, with the exception of one or two, have been removed, while gaps present in only one or two sequences were kept. Figure S7 (RIG-I alignment), Figure S8 (MDA5 alignment) and Figure S9 (LGP2 alignment) correspond to trimmed versions of the nucleotide sequences alignment of each RLR gene.

### Phylogenetic reconstruction analyses

The nucleotide sequences alignment of each gene was firstly tested for recombination, as this biological process can mislead phylogenetic and positive selection analyses [83]. We used the software GARD (Genetic Algorithm for Recombination Detection) [50,51], implemented in the Datamonkey web server [52,53], to detect possible recombination breakpoints on each alignment. The nucleotide substitution model for each phylogenetic reconstruction was indicated by the Akaike Information Criterion (AIC) implemented in jModelTest v0.1.1 [84]. 

Regarding the RIG-I gene one breakpoint was identified, but it was not supported by the Kishino-Hasegawa test. Therefore, the complete alignment was used for the gene phylogeny reconstruction and GTR+G nucleotide substitution model was indicated as the best-fitting model. On the other hand, the software GARD found evidence of two breakpoints in the MDA5 gene alignment. However, only one of the breakpoints (nucleotide 903) reflected a significant topological incongruence (Kishino-Hasegawa test, p<0.01), suggesting that the multiple tree model can be preferred over the single tree model. We reconstructed MDA5 phylogeny for the first 903 nucleotides of the mammalian alignment as also for the remaining 2211 nucleotides. To compare the different MDA5 trees topology, we also used the complete alignment (no recombination testing) to reconstruct the gene phylogeny and GTR+G nucleotide substitution model was indicated by the AIC as the best-fit. For the MDA5 segments which resulted from recombination detection, the best-fitting nucleotide substitution models were TIM3+G (first segment) and TIM3+I+G (second segment). Finally, we found no evidence of recombination for the LGP2 gene alignment. The best-fitting nucleotide substitution model determined for this alignment was the TPM2uf+I+G model.

ML phylogenetic reconstruction was performed for the three genes using GARLI v2.0 (Genetic Algorithm for Rapid Likelihood Inference) [85]. The analyses were performed with 1,000,000 generations and 1,000 bootstrap searches. ML trees were displayed using FigTree v1.3.1 (http://tree.bio.ed.ac.uk/).

### Molecular evolutionary analyses

Codon substitution models implemented in the CODEML program in PAML v4.4 (Phylogenetic Analysis by Maximum Likelihood) package [54,55] were applied to the trimmed alignments of RIG-I (Figure S7), MDA5 (Figure S8) and LGP2 (Figure S9). The codon frequency model F3x4 was fitted to all the alignments. Two pairs of site-specific models were used, M1a (nearly neutral) versus M2a (selection) and M7 (neutral, beta) versus M8 (selection, beta & ω). In these comparisons, M1a and M7 neutral models (null hypothesis) do not admit positive selection, while M2a and M8 alternative models allow positive selection. A LRT with 2 degrees of freedom was performed, where a significant LRT demonstrates that the selection model fits better than the neutral model [56,86,87]. From the HyPhy software available on the Datamonkey web server [52,53], the PARRIS method [57] was also applied to detect if a proportion of sites in each RLR alignment evolved with ω (d_N_/d_S_) > 1. 

Six different codon-based ML methods were applied to detect codons under positive selection on mammalian RIG-I, MDA5 and LGP2 trimmed alignments, and based on the methodology adopted by other authors and in previous studies [43,47,64], only codons identified by at least three of the six used methods were considered to be under positive selection. Model M8 from PAML package [54,55] was one of the codon-based methods used to detect codons under positive selection, and a Bayes empirical Bayes (BEB) approach was employed to detect codons with a posterior probability >90% of being under selection [88]. Five other methods, using HyPhy software implemented in the Datamonkey web server [52,53], were also applied to detect sites under selection for the three genes: the Single Likelihood Ancestor Counting (SLAC) method, the Fixed Effect Likelihood (FEL) method, the Random Effect Likelihood (REL) method [61] and the recently described Mixed Effects Model of Evolution (MEME) [62] and Fast Unbiased Bayesien AppRoximation (FUBAR) [63] methods. To avoid a high false-positive rate [61], sites with *p*-values <0.1 for SLAC, FEL and MEME models, Bayes Factor >50 for REL model and a posterior probability >0.90 for FUBAR were accepted as candidates for selection. 

Two branch-site models allowing ω ratios to vary both among lineages and amino acid sites were performed: the PAML branch-site model A [66] and the Hyphy branch-site REL method [67]. When performing PAML branch-site model A [66], every species branch was analyzed as a foreground branch independently. For each analysis of a foreground branch, the remaining lineages were denominated as background branches. In branch-site model A, three ω ratios are assumed for foreground (0 < *ω*_0_ < 1, *ω*_1_ = 1, *ω*_2_ > 1) and two ω ratios for background (0 < *ω*_0_ < 1, *ω*_1_ = 1). The null model is the same as model A, but *ω*_2_ = 1 is fixed [66]. The BEB approach was also used to calculate the posterior probability of a specific codon site and to identify those most likely to be under positive selection (posterior probability >90%) [88]. On the other hand, the branch-site REL method [67] was applied to identify branches where a proportion of sites evolved under episodic diversifying selection.

## Supporting Information

Figure S1 **Mammalian RIG-I nucleotide coding region sequences alignment.** RIG-I nucleotide coding region sequences for twenty-six mammalian species were collected from Ensembl and NCBI databases, and aligned with ClustalW implemented in BioEdit. The symbol “.” represents the same nucleotide as the reference sequence of human RIG-I gene, “?” symbolizes an undetermined nucleotide and “-“ represents a gap or deletion in the alignment. The used abbreviations correspond, by order of appearance, to the following species: Hosa – Human; Patr – Chimpanzee; Papa – Bonobo; Gogo – Gorilla; Poab – Orangutan; Paan – Olive baboon; Mamu – Rhesus macaque; Sabo – Black-capped squirrel monkey; Caja – Marmoset; Mimu – Mouse lemur; Otga – Bushbaby; Bota – Cow; Ovar – Sheep; Susc – Pig; Mylu – Little brown myotis; Ptva – Large flying fox; Ptal – Black flying fox; Aime – Giant panda; Calu – Dog; Feca – Cat; Eqca – Horse; Loaf – Elephant; Ictr – Squirrel; Capo – Guinea pig; Mumu – Mouse; Orcu – European rabbit.
(TIF)

Figure S2 **Mammalian MDA5 nucleotide coding region sequences alignment.** MDA5 nucleotide coding region sequences for twenty-six mammalian species were collected from Ensembl and NCBI databases, and aligned with ClustalW implemented in BioEdit. The symbol “.” represents the same nucleotide as the reference sequence of human MDA5 gene, “?” symbolizes an undetermined nucleotide and “-“ represents a gap or deletion in the alignment. The used abbreviations correspond, by order of appearance, to the following species: Hosa – Human; Gogo – Gorilla; Patr – Chimpanzee; Papa – Bonobo; Poab – Orangutan; Nole – Gibbon; Mamu – Rhesus macaque; Sabo – Black-capped squirrel monkey; Caja – Marmoset; Otga – Bushbaby; Bota – Cow; Ovar – Sheep; Susc – Pig; Mupu – Ferret; Aime – Giant panda; Calu – Dog; Eqca – Horse; Mylu – Little brown myotis; Ptal – Black flying fox; Loaf – Elephant; Orcu – European rabbit; Crgr – Chinese hamster; Mumu – Mouse; Rano – Rat; Ictr – Squirrel; Capo – Guinea pig.
(TIFF)

Figure S3 **Mammalian LGP2 nucleotide coding region sequences alignment.** LGP2 nucleotide coding region sequences for thirty mammalian species were collected from Ensembl and NCBI databases, and aligned with ClustalW implemented in BioEdit. The symbol “.” represents the same nucleotide as the reference sequence of human LGP2 gene and “-“ symbolizes a gap or deletion in the alignment. The used abbreviations correspond, by order of appearance, to the following species: Hosa – Human; Patr – Chimpanzee; Papa – Bonobo; Gogo – Gorilla; Poab – Orangutan; Mamu – Rhesus macaque; Sabo – Black-capped squirrel monkey; Caja – Marmoset; Mimu – Mouse lemur; Otga – Bushbaby; Bota – Cow; Ovar – Sheep; Susc – Pig; Tutr – Dolphin; Mylu – Little brown myotis; Ptva – Large flying fox; Ptal – Black flying fox; Loaf – Elephant; Mupu – Ferret; Aime – Giant panda; Calu – Dog; Feca – Cat; Eqca – Horse; Ocpr – American pika; Orcu – European rabbit; Ictr – Squirrel; Crgr – Chinese hamster; Mumu – Mouse; Rano – Rat; Capo – Guinea pig.
(TIF)

Figure S4 **Mammalian RIG-I deduced protein sequences alignment.** RIG-I deduced protein sequences for twenty-six mammalian species were collected from Ensembl and NCBI databases, and aligned with ClustalW implemented in BioEdit. The symbol “.” represents the same codon as the reference sequence of human RIG-I protein, “?” symbolizes an undetermined codon and “-“ represents a gap or deletion in the alignment. The used abbreviations correspond, by order of appearance, to the following species: Hosa – Human; Patr – Chimpanzee; Papa – Bonobo; Gogo – Gorilla; Poab – Orangutan; Paan – Olive baboon; Mamu – Rhesus macaque; Sabo – Black-capped squirrel monkey; Caja – Marmoset; Mimu – Mouse lemur; Otga – Bushbaby; Bota – Cow; Ovar – Sheep; Susc – Pig; Mylu – Little brown myotis; Ptva – Large flying fox; Ptal – Black flying fox; Aime – Giant panda; Calu – Dog; Feca – Cat; Eqca – Horse; Loaf – Elephant; Ictr – Squirrel; Capo – Guinea pig; Mumu – Mouse; Orcu – European rabbit.
(TIFF)

Figure S5 **Mammalian MDA5 deduced protein sequences alignment.** MDA5 deduced protein sequences for twenty-six mammalian species were collected from Ensembl and NCBI databases, and aligned with ClustalW implemented in BioEdit. The symbol “.” represents the same codon as the reference sequence of human MDA5 protein, “?” symbolizes an undetermined codon and “-“ represents a gap or deletion in the alignment. The used abbreviations correspond, by order of appearance, to the following species: Hosa – Human; Gogo – Gorilla; Patr – Chimpanzee; Papa – Bonobo; Poab – Orangutan; Nole – Gibbon; Mamu – Rhesus macaque; Sabo – Black-capped squirrel monkey; Caja – Marmoset; Otga – Bushbaby; Bota – Cow; Ovar – Sheep; Susc – Pig; Mupu – Ferret; Aime – Giant panda; Calu – Dog; Eqca – Horse; Mylu – Little brown myotis; Ptal – Black flying fox; Loaf – Elephant; Orcu – European rabbit; Crgr – Chinese hamster; Mumu – Mouse; Rano – Rat; Ictr – Squirrel; Capo – Guinea pig.
(TIFF)

Figure S6 **Mammalian LGP2 deduced protein sequences alignment.** LGP2 deduced protein sequences for thirty mammalian species were collected from Ensembl and NCBI databases, and aligned with ClustalW implemented in BioEdit. The symbol “.” represents the same codon as the reference sequence of human LGP2 protein and “-“ symbolizes a gap or deletion in the alignment. The used abbreviations correspond, by order of appearance, to the following species: Hosa – Human; Patr – Chimpanzee; Papa – Bonobo; Gogo – Gorilla; Poab – Orangutan; Mamu – Rhesus macaque; Sabo – Black-capped squirrel monkey; Caja – Marmoset; Mimu – Mouse lemur; Otga – Bushbaby; Bota – Cow; Ovar – Sheep; Susc – Pig; Tutr – Dolphin; Mylu – Little brown myotis; Ptva – Large flying fox; Ptal – Black flying fox; Loaf – Elephant; Mupu – Ferret; Aime – Giant panda; Calu – Dog; Feca – Cat; Eqca – Horse; Ocpr – American pika; Orcu – European rabbit; Ictr – Squirrel; Crgr – Chinese hamster; Mumu – Mouse; Rano – Rat; Capo – Guinea pig.
(TIF)

Figure S7 **Mammalian RIG-I nucleotide trimmed sequences alignment.** RIG-I nucleotide trimmed sequences for twenty-six mammalian species were collected from Ensembl and NCBI databases, and aligned with ClustalW implemented in BioEdit. The symbol “.” represents the same nucleotide as the reference sequence of human RIG-I gene, “?” symbolizes an undetermined nucleotide and “-“ represents a gap or deletion in the alignment. The used abbreviations correspond, by order of appearance, to the following species: Hosa – Human; Patr – Chimpanzee; Papa – Bonobo; Gogo – Gorilla; Poab – Orangutan; Paan – Olive baboon; Mamu – Rhesus macaque; Sabo – Black-capped squirrel monkey; Caja – Marmoset; Mimu – Mouse lemur; Otga – Bushbaby; Bota – Cow; Ovar – Sheep; Susc – Pig; Mylu – Little brown myotis; Ptva – Large flying fox; Ptal – Black flying fox; Aime – Giant panda; Calu – Dog; Feca – Cat; Eqca – Horse; Loaf – Elephant; Ictr – Squirrel; Capo – Guinea pig; Mumu – Mouse; Orcu – European rabbit.
(TIF)

Figure S8 **Mammalian MDA5 nucleotide trimmed sequences alignment.** MDA5 nucleotide trimmed sequences for twenty-six mammalian species were collected from Ensembl and NCBI databases, and aligned with ClustalW implemented in BioEdit. The symbol “.” represents the same nucleotide as the reference sequence of human MDA5 gene, “?” symbolizes an undetermined nucleotide and “-“ represents a gap or deletion in the alignment. The used abbreviations correspond, by order of appearance, to the following species: Hosa – Human; Gogo – Gorilla; Patr – Chimpanzee; Papa – Bonobo; Poab – Orangutan; Nole – Gibbon; Mamu – Rhesus macaque; Sabo – Black-capped squirrel monkey; Caja – Marmoset; Otga – Bushbaby; Bota – Cow; Ovar – Sheep; Susc – Pig; Mupu – Ferret; Aime – Giant panda; Calu – Dog; Eqca – Horse; Mylu – Little brown myotis; Ptal – Black flying fox; Loaf – Elephant; Orcu – European rabbit; Crgr – Chinese hamster; Mumu – Mouse; Rano – Rat; Ictr – Squirrel; Capo – Guinea pig.
(TIFF)

Figure S9 **Mammalian LGP2 nucleotide trimmed sequences alignment.** LGP2 nucleotide trimmed sequences for thirty mammalian species were collected from Ensembl and NCBI databases, and aligned with ClustalW implemented in BioEdit. The symbol “.” represents the same nucleotide as the reference sequence of human LGP2 gene and “-“ symbolizes a gap or deletion in the alignment. The used abbreviations correspond, by order of appearance, to the following species: Hosa – Human; Patr – Chimpanzee; Papa – Bonobo; Gogo – Gorilla; Poab – Orangutan; Mamu – Rhesus macaque; Sabo – Black-capped squirrel monkey; Caja – Marmoset; Mimu – Mouse lemur; Otga – Bushbaby; Bota – Cow; Ovar – Sheep; Susc – Pig; Tutr – Dolphin; Mylu – Little brown myotis; Ptva – Large flying fox; Ptal – Black flying fox; Loaf – Elephant; Mupu – Ferret; Aime – Giant panda; Calu – Dog; Feca – Cat; Eqca – Horse; Ocpr – American pika; Orcu – European rabbit; Ictr – Squirrel; Crgr – Chinese hamster; Mumu – Mouse; Rano – Rat; Capo – Guinea pig.
(TIF)

Table S1 **List of mammalian species and gene accession numbers used in this study.**
(PDF)

Table S2 **RIG-I, MDA5 and LGP2 likelihood ratio test (LRT) for PARRIS analysis from HyPhy software.**
(PDF)

Table S3 **Positively-selected codon positions for RIG-I, MDA5 and LGP2 determined by six different methods.**
(PDF)
