# Supplementary material for: Positive Evolutionary Selection On the RIG-I-Like Receptor Genes in Mammals
Source: PLoS One. 2013 Nov 27;8(11):e81864. doi: 10.1371/journal.pone.0081864 (PMC3842351; doi:10.1371/journal.pone.0081864)
Supplement: Table S1 — List of mammalian species and gene accession numbers used in this study. (PDF) [file pone.0081864.s010.pdf]

Order	Family	Species common name (*Scientific name*)	Gene_Abbreviation	Database ID
Artiodactyla	Bovidae	Cow (*Bos taurus*)	LGP2_Bota	ENSBTAT00000065738 [a]
			MDA5_Bota	ENSBTAG00000008142 [a]
			RIG-I_Bota	ENSBTAG00000003366 [a]
		Sheep (*Ovis aries*)	LGP2_Ovar	XM_004012918.1 [b]
			MDA5_Ovar	XM_004004655.1 [b]
			RIG-I_Ovar	XM_004005323.1 [b]
	Suidae	Pig (*Sus scrofa*)	LGP2_Susc	ENSSSCT00000018960 [a]
			MDA5_Susc	NM_001100194.1 [b]
			RIG-I_Susc	ENSSSCG00000030408 [a]
Carnivora	Canidae	Dog (*Canis lupus familiaris*)	LGP2_Calu	ENSCAFG00000015720 [a]
			MDA5_Calu	ENSCAFG00000010438 [a]
			RIG-I_Calu	ENSCAFG00000001807 [a]
	Felidae	Cat (*Felis catus*)	LGP2_Feca	XM_003996876.1 [b]
			RIG-I_Feca	XM_003995540.1 [b]
	Mustelidae	Ferret (*Mustela putorius furo*)	LGP2_Mupu	ENSMPUG00000010568 [a]
			MDA5_Mupu	ENSMPUG00000000564 [a]
	Ursidae	Giant panda (*Ailuropoda melanoleuca*)	LGP2_Aime	ENSAMEG00000005941 [a]
			MDA5_Aime	ENSAMEG00000005698 [a]
			RIG-I_Aime	ENSAMEG00000003766 [a]
Cetacea	Delphinidae	Dolphin (*Tursiops truncatus*)	LGP2_Tutr	ENSTTRG00000006650 [a]
Chiroptera	Pteropodidae	Black flying fox (*Pteropus alecto*)	LGP2_Ptal	JN031516.1 [b]
			MDA5_Ptal	JN031515.1 [b]
			RIG-I_Ptal	JN031514.1 [b]
		Large flying fox (*Pteropus vampyrus*)	LGP2_Ptva	ENSPVAG00000003855 [a]
			RIG-I_Ptva	ENSPVAG00000009207 [a]
	Vespertilionidae	Little brown myotis (*Myotis lucifugus*)	LGP2_Mylu	ENSMLUG00000012001 [a]
			MDA5_Mylu	ENSMLUG00000009505 [a]
			RIG-I_Mylu	ENSMLUG00000003041 [a]
Perissodactyla	Equidae	Horse (*Equus caballus*)	LGP2_Eqca	XM_001495212.2 [b]
			MDA5_Eqca	ENSECAT00000008541 [a]
			RIG-I_Eqca	ENSECAG00000021989 [a]
Proboscidea	Elephantidae	Elephant (*Loxodonta africana*)	LGP2_Loaf	ENSLAFG00000027301[a]
			MDA5_Loaf	XM_003405827.1[b]
			RIG-I_Loaf	ENSLAFG00000005416 [a]
Lagomorpha	Leporidae	European rabbit (*Oryctolagus cuniculus*)	LGP2_Orcu	ENSOCUG00000013278 [a]
			MDA5_Orcu	ENSOCUG00000002863 [a]
			RIG-I_Orcu	ENSOCUT00000029633 [a]
	Ochotonidae	American pika (*Ochotona princeps*)	LGP2_Ocpr	ENSOPRG00000000753 [a]
Primates	Cebidae	Black-capped squirrel monkey (*Saimiri boliviensis boliviensis*)	LGP2_Sabo	XM_003942770.1 [b]
			MDA5_Sabo	XM_003921929.1 [b]
			RIG-I_Sabo	XM_003939729.1 [b]
		Marmoset (*Callithrix jacchus*)	LGP2_Caja	ENSCJAT00000028952 [a]
			MDA5_Caja	ENSCJAT00000011621 [a]
			RIG-I_Caja	ENSCJAT00000015302 [a]
	Cercopithecidae	Olive baboon (*Papio anubis*)	RIG-I_Paan	XM_003911615.1 [b]
		Rhesus macaque (*Macaca mulatta*)	LGP2_Mamu	ENSMMUG00000014755 [a]
			MDA5_Mamu	ENSMMUG00000003202 [a]
			RIG-I_Mamu	ENSMMUT00000017446 [a]
	Cheirogaleidae	Mouse lemur (*Microcebus murinus*)	LGP2_Mimu	ENSMICG00000011933 [a]
			RIG-I_Mimu	ENSMICG00000006555 [a]
	Galagidae	Bushbaby (*Otolemur garnettii*)	LGP2_Otga	ENSOGAG00000025321 [a]
			MDA5_Otga	ENSOGAG00000012611 [a]
			RIG-I_Otga	ENSOGAG00000010536 [a]
	Hominidae	Bonobo (*Pan paniscus*)	LGP2_Papa	XM_003813868.1 [b]
			MDA5_Papa	XM_003820935.1 [b]
			RIG-I_Papa	XM_003830100.1 [b]
		Chimpanzee (*Pan troglodytes*)	LGP2_Patr	ENSPTRG00000009191 [a]
			MDA5_Patr	ENSPTRG00000012582 [a]
			RIG-I_Patr	ENSPTRG00000020844 [a]
		Human (*Homo sapiens*)	LGP2_Hosa	ENST00000251642 [a]
			MDA5_Hosa	ENST00000263642 [a]
			RIG-I_Hosa	ENST00000379883 [a]
		Orangutan (*Pongo abelii*)	LGP2_Poab	NM_001131127.1 [b]
			MDA5_Poab	ENSPPYG00000012887 [a]
			RIG-I_Poab	XM_002819715.1 [b]
		Gorilla (*Gorilla gorilla*)	LGP2_Gogo	ENSGGOG00000005458 [a]
			MDA5_Gogo	ENSGGOT00000015950 [a]
			RIG-I_Gogo	ENSGGOT00000032102 [a]
	Hylobatidae	Gibbon (*Nomascus leucogenys*)	MDA5_Nole	XM_003266320.1 [b]
Rodentia	Caviidae	Guinea pig (*Cavia porcellus*)	LGP2_Capo	ENSCPOG00000023721 [a]
			MDA5_Capo	ENSCPOG00000007154 [a]
			RIG-I_Capo	ENSCPOG00000001598 [a]
	Cricetidae	Chinese hamster (*Cricetulus griseus*)	LGP2_Crgr	XM_003504640.1 [b]
			MDA5_Crgr	XM_003508631.1 [b]
	Muridae	Rat (*Rattus norvegicus*)	LGP2_Rano	ENSRNOG00000018247 [a]
			MDA5_Rano	ENSRNOG00000006227 [a]
		Mouse (*Mus musculus*)	LGP2_Mumu	ENSMUST00000017974 [a]
			MDA5_Mumu	ENSMUST00000028259 [a]
			RIG-I_Mumu	ENSMUST00000037907[a]
	Sciuridae	Squirrel (*Ictidomys tridecemlineatus*)	LGP2_Ictr	ENSSTOG00000006524 [a]
			MDA5_Ictr	ENSSTOG00000004953 [a]
			RIG-I_Ictr	ENSSTOG00000006994 [a]

Database ID: [a]Ensembl; [b]NCBI GenBank
