# Supplementary material for: Positive Evolutionary Selection On the RIG-I-Like Receptor Genes in Mammals
Source: PLoS One. 2013 Nov 27;8(11):e81864. doi: 10.1371/journal.pone.0081864 (PMC3842351; doi:10.1371/journal.pone.0081864)
Supplement: Table S2 — RIG-I, MDA5 and LGP2 likelihood ratio test (LRT) for PARRIS analysis from HyPhy software. (PDF) [file pone.0081864.s011.pdf]

PARRIS analysis	lnL_{null} [a]	$lnL_{alternative}$ [a]	$2\Delta lnL$ [b]	*p*-Value	M2 parameters estimate
**RIG-I**					ω_1 = 0.07 (p = 0.571)
M1 $_{(no\ selection)}$ vs. M2 $_{(selection)}$	-20727.6	-20727.6	0	n.s.	ω_2 = 1.00 (p = 0.429)
					ω_3 = 4.57 (p = 0.000)
**MDA5**					ω_1 = 0.06 (p = 0.641)
M1 $_{(no\ selection)}$ vs. M2 $_{(selection)}$	-21908.5	-21908.5	0	n.s.	ω_2 = 1.00 (p = 0.359)
					ω_3 = 4.43 (p = 0.000)
**LGP2**					ω_1 = 0.08 (p = 0.740)
M1 $_{(no\ selection)}$ vs. M2 $_{(selection)}$	-18390.7	-18390.7	0	n.s.	ω_2 = 1.00 (p = 0.260)
					ω_3 = 5.16 (p = 0.000)

[a] lnL: log-likelihood scores.
[b] $2\Delta lnL$: likelihood ratio test (LRT) to detect positive selection.
n.s. – non-significant
