# Supplementary material for: Positive Evolutionary Selection On the RIG-I-Like Receptor Genes in Mammals
Source: PLoS One. 2013 Nov 27;8(11):e81864. doi: 10.1371/journal.pone.0081864 (PMC3842351; doi:10.1371/journal.pone.0081864)
Supplement: Table S3 — Positively-selected codon positions for RIG-I, MDA5 and LGP2 determined by six different methods. (PDF) [file pone.0081864.s012.pdf]

	RIG-I	MDA5	LGP2
**PAML M8**[a]	10*, 13*, 31, **62**, **80***, 107*, 123, 142*, 183*, 196*, 201*, **224***, 241*, **245**, 528*, 534*, 585*, 601*, 610, 703, **783**, **800**, **808***, **816***, 871*, **874***, 920, 944*	**59**, **846**, 1008*	**74**, **180***, 269*, 324*, 347*, **438****, **627***, 653*
**SLAC**[b]	**250**, **337**, **783***, **800**, **808**, **816**, **874***	**11**, **53***, **59***, **212***, **218**, **305**, **395**, **533***, **673**, **684**, **871**, **909***, **1002**	**97**, **145***, **180**, **205***, **316****, **438**, 554, **618***, 674
**FEL**[b]	5, **35***, **61***, **62***, **80**, 97, **135***, 208, **224***, **240***, **245***, **250***, **337***, 467, 509*, 510, **516**, 535, 630*, 681, 684, 768, **783***, **800***, 805, **808****, **816***, 823*, 858, **874****, 878, 889	**11***, 17, **53****, **59****, **73****, 142, **144***, **149**, **156**, 172, **197***, 204, **212****, **218***, **242***, 252, **305***, 307, 348, 385, **395**, 486, 523, **533****, 672*, **673**, **684***, 713, **846***, **871***, 907*, **909****, **1002***	47, **97**, **145***, **180***, 197, **205***, 233, **312**, **316****, **438****, **618***, **627**, 658
**REL**[c]	**35**, **61**, **62**, **80**, **135**, **224**, **240**, **245**, 308, **337**, **516**, **783**, **800**, **808**, **816**, **874**	**11**, 17, 24, **53**, **59**, **73**, **144**, **149**, **156**, **197**, 204, **212**, **218**, **242**, 302, 303, **305**, 414, **533**, 649, 671, **673**, **684**, 688, 713, **846**, **871**, **909**, **1002**, 1008, 1033	**74**, **97**, **145**, **180**, **205**, **312**, **316**, **438**, **618**
**MEME**[b]	5, 21*, **35***, 44, **61***, **62***, **80**, 97*, 98, 121*, 123*, **135**, 150*, 195*, 196**, 198, 211, **224**, 232*, 236, **240****, 242**, **245***, 246**, **250****, 252*, 259*, 262**, **337***, 407*, 467*, 478**, 481, 492, 497, 500, 509*, 510, **516***, 528, 535*, 630, 655**, 656*, 667, 669**, 681, 684*, 696*, 768, 772, 779, **783***, **800***, 805, **808***, 814*, **816***, 818, 823, 849, 851, 858*, 865, 870*, 873**, **874***, 889*, 926, 940**, 942**	**11****, 26, 42, **53***, **59****, **73***, 142*, **144***, **156**, 195, **197**, **212****, **218****, 228, 237*, **242**, 303, **305***, 307*, 348*, 374, 385*, 386, **395**, 437*, 440*, 482*, **533***, 551**, 566, 589, 593*, 594**, 595, 599*, 641, **673***, **684***, 688, 691, **846***, **871***, 907, **909***, 967*, 995**, **1002**, 1004, 1032*	47*, **74***, **97**, 125*, 130, **145***, **180***, **205***, 233, 253*, 272, 276, 301, **312**, **316***, 318, 321, 327, 368, 401, 405*, 406*, 416, **438****, 519**, 520*, 569, 579*, 594**, 609**, **618***, **627**, 637*, 674
**FUBAR**[d]	**62**, **80**, **224**, **245**, **783**, **800**, **808***, **816**, **874***	**53***, **59***, **73***, **149**, **212**, **305**, **533***, 649, **673**, **846**, **909***, **1002***	**180***, **205**, **316**, **438****, **618**, **627***

[a]Sites with posterior probabilities >0.90 in the BEB (Bayes Empirical Bayes) analyses (*: P>0.95; **: P>0.99).

[b]Sites with significance level <0.1 (*: p<0.05; **: p<0.01).

[c]Sites with Bayes Factor >50.

[d]Sites with posterior probabilities >0.90 (*: P>0.95; **: P>0.99).

Sites identified by three or more than three methods are in bold.
